# Sex differences in Huntington's disease from a neuroinflammation perspective

**DOI:** 10.3389/fneur.2024.1384480

**Published:** 2024-06-10

**Authors:** Grace Risby-Jones, John D. Lee, Trent M. Woodruff, Jenny N. Fung

**Affiliations:** ^1^School of Biomedical Sciences, The University of Queensland, St Lucia, QLD, Australia; ^2^Queensland Brain Institute, The University of Queensland, St Lucia, QLD, Australia

**Keywords:** Huntington (disease), neuroinflammation, sex difference, microglia, oligodendrocytes (OLs), astrocytes

## Abstract

Huntington's disease (HD) is a debilitating neurodegenerative condition characterized by motor, cognitive and psychiatric abnormalities. Immune dysregulation, prominently featuring increased immune activity, plays a significant role in HD pathogenesis. In addition to the central nervous system (CNS), systemic innate immune activation and inflammation are observed in HD patients, exacerbating the effects of the Huntingtin (HTT) gene mutation. Recent attention to sex differences in HD symptom severity underscores the need to consider gender as a biological variable in neurodegenerative disease research. Understanding sex-specific immune responses holds promise for elucidating HD pathophysiology and informing targeted treatment strategies to mitigate cognitive and functional decline. This perspective will highlight the importance of investigating gender influence in HD, particularly focusing on sex-specific immune responses predisposing individuals to disease.

## Introduction

Huntington's disease (HD) is the most common inherited neurodegenerative disease, characterized by uncontrolled choreatic movements, behavioral and psychiatric disturbances, and dementia (1–3). While symptoms typically emerge in middle age, they can manifest at any point from infancy to old age (3). The mutation responsible for HD is an expanded cytosine-adenine-guanine (CAG) trinucleotide repeat in the huntingtin (Htt) gene leading to an abnormally long polyglutamine tail that confers a toxic gain of function (1, 3). As it is an autosomal dominant inherited disease, there is an equal penetrance and prevalence in men and women in the population (4). Notably however, recent findings from a large American cohort study comprising 3,707 HD individuals revealed a slightly higher prevalence among females compared to males (7.05 vs. 6.10 per 100,000 persons, respectively), suggesting a possible relationship between sex and HD (5). Moreover, evidence from various animal models and epidemiologic cohorts suggests that sex may contribute to the variability in disease manifestation between men and women with HD (4).

## Sex differences in HD

Sex differences in HD are multifaceted, encompassing various aspects of disease progression and symptomatology. Notably, its role in disease anticipation has been observed, with paternal transmission resulting in earlier onset and faster progression compared to maternal transmission due to CAG repeat instability primarily during spermatogenesis (6, 7). Consequently, large expansions in CAG repeats predominantly occur through paternal transmission, while offspring of affected mothers are more likely to show no change or contractions in CAG length (8). Clinical observations from large-scale studies, such as the REGISTRY (European Huntington's Disease Network) and ENROLL-HD databases, consistently report that females with HD present with more symptoms, exhibit a slightly more severe phenotype, and experience a faster rate of progression, particularly in motor and functional domains (9, 10). Moreover, another study based on the REGISTRY database found that motor symptoms have a more pronounced impact on functional ability and independence in women with HD compared to men (7, 11, 12). Furthermore, analyses of the PREDICT-HD using REGISTRY and ENROLL-HD databases demonstrated that depression is more prevalent and severe in females with HD (9, 10, 13).

Sex hormones, for example testosterone, have also been suggested to have neuroprotective effects. Reduced plasma testosterone levels in males with HD correlate with increased disease severity and cognitive impairment (14), while decreased plasma testosterone levels in females are associated with depression (6, 11, 15). In contrast to human studies, animal studies suggest that estrogen exerts neuroprotective effects against mechanisms underlying HD development, specifically oxidative stress and glutamate excitotoxicity (4, 16). Specifically, 17β-estradiol has been shown to protect against markers of oxidative stress in models of HD, with females exhibiting less severe motor dysfunction compared to males (17, 18). However, it remains unclear which sex hormones are neuroprotective and which are neurotoxic. More research is needed to understand the role of sex hormones in HD pathologies.

However, contradicting results regarding sex-related differences in HD have been reported. A study conducted by the Huntington Study Group found no significant sex-related effects on function, motor, cognitive and behavioral domains in a large cohort of HD patients (6, 19). While hormonal differences may not be the direct contributor of the sex-specific symptoms or disease progression, sex steroids can regulate the transcription of genes relevant to immune cell development, immune responses, and immune signaling pathways (20). Emerging evidence suggests that immune cell size, morphology, and function, as well as glial cells and inflammation markers may exhibit sex-specific characteristics, highlighting the intricate interplay between sex hormones, immune responses, and HD pathology.

## Neuroinflammation in HD

Neuroinflammation is a major hallmark for HD and is characterized by the reactive morphology of glial cells: microglia, astrocytes, and oligodendrocytes. These cells are vital for regulating neuronal activity and maintaining optimal neuronal function (21). Despite their crucial roles, activated microglia and reactive astrocytes have been implicated in HD pathology, perpetuating a chronic inflammatory state by upregulating proinflammatory genes, ultimately, contributing to neuronal death observed in HD progression (21).

Microglia expressing mutant Htt (mHtt) in HD play a neurotoxic role by inducing a proinflammatory response, characterized by increased expression of Interleukin (IL)-6, Tumor Necrosis Factor (TNF)-α and reactive oxygen species (ROS) (22). Similarly, reactive astrocytes in HD produce proinflammatory and harmful molecules such as TNF-α, IL-6, ROS, and mHtt aggregates, exacerbating neuroinflammation (22). Dysfunction of astrocyte in HD, marked by deficits in calcium signaling, iron homeostasis and neurotransmitter clearance, further contributes to disease pathology (22, 23).

**Figure 1:**
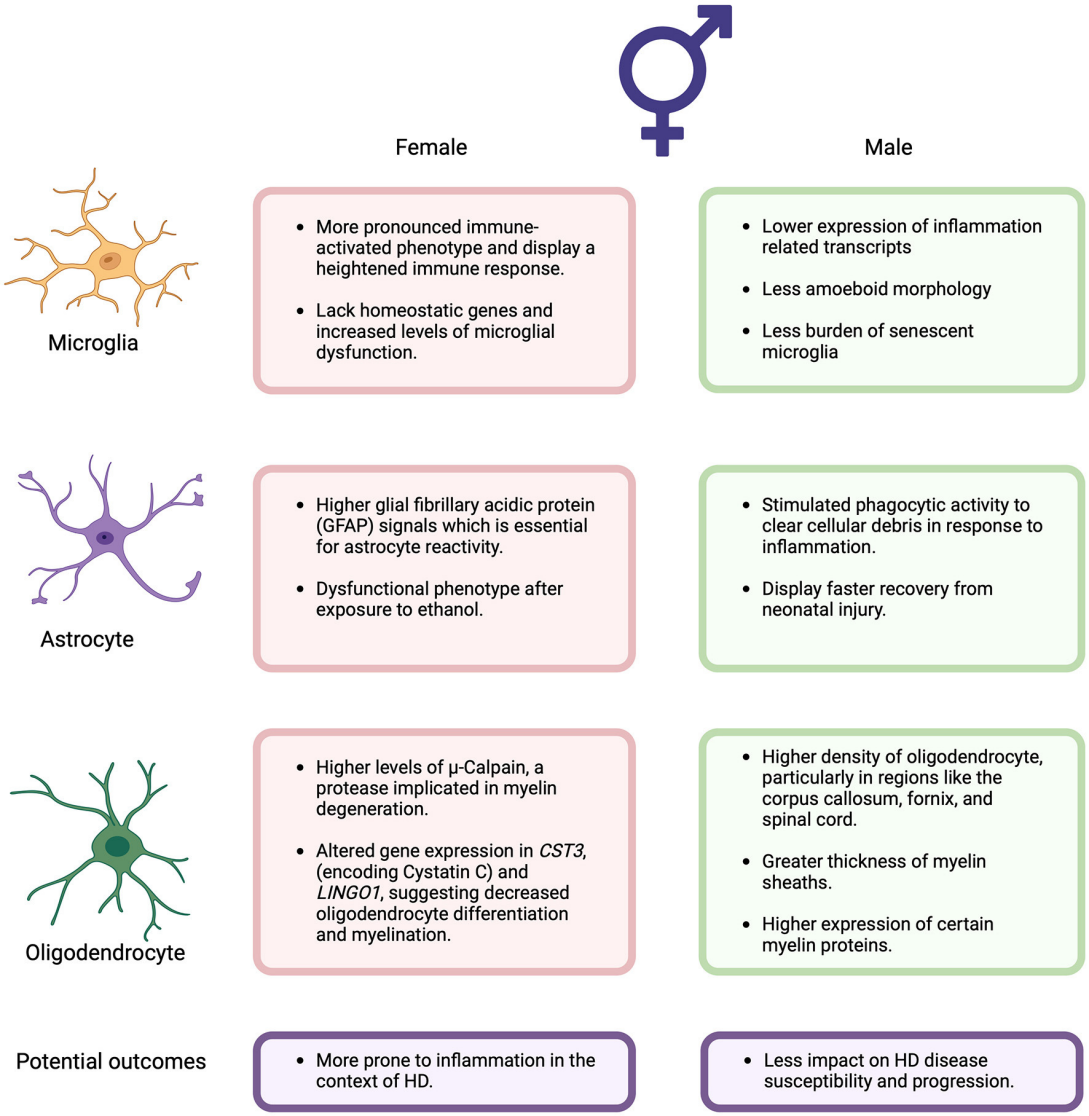
Summary of the sex differences in the potential functional roles of microglia, astrocytes, and oligodendrocytes associated with Huntington's Disease discussed in this perspective. Figure created using BioRender (Biorender.com).

Oligodendrocytes are the myelinating glia of the CNS that play crucial roles in rapid transmission of electrical signals between neurons and providing trophic support for neurons (24–26). In mouse models of HD, early structural impairments in myelin have been observed, accompanied by significant reductions in the expression of myelin-related genes in striatal and cortical tissues, as well as isolated oligodendrocytes from HD mice (24, 27–30). These myelination deficits likely impair the transmission of information across neuronal circuits, as seen by motor performance impairments in mice expressing mHTT specifically in oligodendroglia cells and the rescue of various HD-related pathologies when it is genetically excised from HD mice (24, 27, 31). While the functional roles of microglia, astrocytes, and oligodendrocytes in HD progression have been extensively reviewed (32, 33), emerging evidence suggests sex differences in these glial cells may impact neurodegenerative disease outcomes. Here, we focus on the sex-specific role of these cells in HD progression, a topic that has not been extensively discussed previously (Figure 1).

## Sex differences in neuroinflammation

### Sex differences in microglia

Independent of hormonal signals, microglia from female mice have been found to be neuroprotective, limiting the damage caused by acute focal cerebral (34). However, emerging evidence suggests that microglia from mature females adopt a more immune-activated phenotype, consistent with the finding that females have a stronger immune response than males (35–37). This is supported by studies of the aging brain where microglia in females have higher expression of inflammation-related transcripts compared to males (38, 39). Additionally, female rats exhibit a greater proportion of microglia with an amoeboid morphology and thicker processes in specific brain regions such as the hippocampus, amygdala, and parietal cortex (35, 40). Moreover, female mice demonstrate enriched microglia-specific gene expression in the hippocampus relative to males, with higher expression of genes associated with inflammation and apoptosis in female-derived microglia (35, 41). Furthermore, studies indicate that female mice lack homeostatic genes across different life stages (juveniles, adults and in old age), leading to increased levels of microglial dysfunction in aged females compared to males (39, 42). Aged females also possess a greater burden of senescent microglia, which may contribute to the development of neurodegenerative diseases and a more rapid and severe disease course in females (39, 42, 43). While research on sex differences in microglia in HD remains limited compared to other neurodegenerative diseases like Alzheimer's disease (AD), studies in AD have shown that aged female donors display higher expression of gene signatures related to inflammatory response and proinflammatory immune responses in microglia nuclei compared to males (44). Additionally, functional studies involving microglial transplants and analyses of microglial Ca^2+^ signaling and process motility suggest that female mice exhibit more rapid aging of microglia than males (38, 45). These findings indicate that female-derived microglia may be more prone to proinflammation activation and dysfunction, potentially contributing to the progression of neurodegenerative diseases like HD.

### Sex differences in astrocytes

Astrocytes have also been found to have sex differences in their response to inflammation, which may have implications in HD progression. Upregulation of the intermediate filament glial fibrillary acidic protein (GFAP) is essential for the transition of astrocytes to a reactive state (46). A recent study identified a significant elevation in plasma GFAP concentration in HD patients with the HTT mutation, suggesting its association with HD progression (47). Female mice were found to have significantly higher GFAP signals than males (48, 49), indicating that female astrocytes may be more prone to inflammation than males in the context of HD. In contrast, *in vitro* studies have shown that female astrocytes exhibit enhanced expression of anti-inflammatory cytokine, interferon-gamma-inducible protein 10 (IP-10/CXCL10), in response to Lipopolysaccharides (LPS) (50). Whereas male astrocytes show enhanced expression of pro-inflammatory markers including IL-6, TNF-α and IL-1β, after LPS treatment (50). Nevertheless, the sex-dependent changes in astrocytes contributing to neuroprotection or neurotoxicity are complex and still largely unknown.

Recent studies have revealed sex differences in the functional role of astrocyte such as phagocytosis. *In vitro* studies have shown that male-derived astrocytes are stimulated to phagocytose brain-derived cellular debris, to re-establish tissue homeostasis in response to inflammation, whereas phagocytic activity is inhibited in female-derived astrocytes (38, 51). Moreover, phagocytic responses in primary astrocytes obtained from HD mouse models progressively decrease over time (52). Exposure to ethanol induces a dysfunctional phenotype in female astrocytes but not in males (38, 53). Conversely, primary cultures from newborn rats suggest that female astrocytes may exhibit greater resistance to oxygen-glucose deprivation-induced cell death, indicating a better metabolic adaptation response to insults (38, 54). Furthermore after *in vivo* neonatal hypoxia-ischemia, female astrocytes demonstrate enhanced mitochondrial metabolism compared to males (38, 55), although males display faster recovery from the neonatal injury (38, 55). These findings suggest that sex differences in astrocytes may have significant functional consequences for HD progression, particularly in response to non-homeostatic disease states.

### Sex differences in oligodendrocytes

Oligodendrocytes display numerous sex differences in neurodegenerative diseases, potentially impacting disease susceptibility and progression. Firstly, studies in rats have shown higher density of oligodendrocytes in male rats compared to females in regions such as the corpus callosum, fornix and spinal cord (38, 56). This difference is associated with greater thickness of myelin sheaths and higher expression of certain myelin proteins in males, along with a shorter lifespan of female oligodendrocytes (38, 56). Moreover, females exhibit significantly higher transcriptional and translational levels of μ-Calpain, a protease implicated in the degeneration of myelin, compared to males (56). In AD, single-cell transcriptome analyses have revealed sex differences in oligodendrocytes. Males with AD display increased pathology associated with global transcriptional activation in oligodendrocytes, a phenomenon not observed in females (38, 57). Additionally, female oligodendrocyte precursors demonstrate a down-regulation shift in response to pathology that is absent in male cells (38, 57).

Furthermore, a single-cell RNA-seq analysis in AD identified sex differences in gene expression patterns of oligodendrocytes. *CST3*, encoding Cystatin C, is upregulated in neurons and oligodendrocytes of AD males but downregulated in AD females (58). Cystatin C is involved in membrane structure and cellular signaling, contributing to blood brain barrier integrity and reduced permeability (59). Reduced levels of CSF cystatin C have been linked to increased susceptibility to neurodegeneration (60). A recent single-nucleus RNAseq study from HD and control post-mortem brain tissues showed that expression of *CST3* is downregulated in the oligodendrocytes and oligodendrocyte precursors (OPCs) of the HD caudate (61). Thus, as cystatin C is downregulated in female oligodendrocytes, this suggests that females are more vulnerable to AD pathology and likely other neurodegenerative diseases, like HD (58). Additionally, Leucine-rich repeat and immunoglobulin-like domain-containing nogo receptor-interacting protein 1 (*LINGO1*) were found to be upregulated in AD females but not in males (58). LINGO1 is a negative modulator of neuronal survival, axonal integrity and myelination (62, 63). As AD females exhibit upregulated levels of *LINGO1*, they may have decreased oligodendrocyte differentiation and myelination (63). The single-nucleus RNAseq study from HD and control post-mortem brain tissues also showed that expression of *LINGO1* is up-regulated in the oligodendrocytes and OPCs of the HD caudate and cingulate (61). Altogether, these studies indicate that oligodendrocytes differentiation and myelination, as well as the response to pathology, are sex-dependent, with females being more susceptible to these changes and therefore to neurodegenerative diseases, such as HD.

## Potential sex-specific treatment for HD

Sex-specific responses to treatments in HD highlight the importance of considering sex as a biological variable in therapeutic development. While studies have found no sex differences in the safety and efficacy of certain treatments like tetrabenazine, for managing chorea (6, 64–66), there may be variations in dosage and treatment duration between males and females. For example, preclinical studies in zQ175 HD mice have shown that both sexes benefit from mGluR5 negative allosteric modulator treatment, but females required a longer treatment duration for optimal efficacy (6, 67). Regarding the treatment of mood disorders, it has been reported from the PREDICT-HD study that females with HD are more likely to use anxiolytics and antidepressants than males at early disease stages of HD (68), possibly due to greater disease severity and progression.

Emerging evidence suggests that targeting glial cell dysfunction, with consideration for sex-specific differences, may offer novel interventions in treating HD patients. Inhibition of phagocytosis activity in female astrocytes (38, 51), coupled with progressively reduced phagocytic responses in both R6/2 and zQ175 HD mouse models (52), underscores the potential benefits of modulating astrocytic phagocytosis for female HD patients. Tibolone, a synthetic steroid used for menopausal symptoms (69), has shown promise in stimulating phagocytosis activity in both sexes, with greater effect observed in females (38). Importantly, tibolone's mechanism of action varies between male and female cells, indicating potential sex-specific therapeutic strategies (51).

While therapeutic approaches targeting oligodendrocytes in HD are currently lacking, research suggests that females may have decreased oligodendrocyte differentiation and myelination, rendering them potentially more susceptible to HD progression (63). Repurposing existing drugs that promote oligodendrocyte progenitor cell differentiation and myelination, such as clemastine fumarate, could offer therapeutic avenues for HD. Clemastine fumarate has demonstrated myelin repair potential in multiple sclerosis patients, indicating a possible role in slowing neurodegenerative disease progression (70, 71). However, further research is needed to evaluate its efficacy and safety for HD treatment.

## Conclusion

In conclusion, sex differences in HD present a complex and multifaceted landscape. While female HD patients often exhibit a higher prevalence and more severe disease symptoms, contradictory results from animal models and clinical studies underscore the need for deeper investigation. The role of sex differences in neuroinflammation, particularly the heightened proinflammatory response and sensitivity to dysregulation observed in female microglia and oligodrocytes, may contribute to the worsened disease phenotype in females. In contrast, while astrocytes display sex-specific changes, there is limited knowledge about the functional consequences of these changes in neurodegeneration. However, the conflicting nature of existing research highlights the necessity for additional studies to fully elucidate the impact of sex differences in neuroinflammation on HD progression.

Current pharmacological interventions hold promise in modulating glial function, potentially offering sex-specific effects. Nonetheless, a deeper understanding of their mechanisms of action and optimisation for different patient populations is imperative. Future drug development endeavors may benefit from a focus on designing treatments that specifically target glial cells, with careful consideration for sex-specific differences. Ultimately, advancing our understanding of sex differences in HD and developing tailored therapeutic strategies hold the key to improving outcomes for all patients affected by this debilitating neurodegenerative disease.

## Data availability statement

The original contributions presented in the study are included in the article/supplementary material, further inquiries can be directed to the corresponding author.

## Author contributions

GR-J: Data curation, Writing – original draft, Writing – review & editing. JL: Conceptualization, Supervision, Writing – original draft, Writing – review & editing. TW: Funding acquisition, Supervision, Writing – original draft, Writing – review & editing. JF: Conceptualization, Data curation, Supervision, Visualization, Writing – original draft, Writing – review & editing.
